# IHC-based Ki67 as response biomarker to tamoxifen in breast cancer window trials enrolling premenopausal women

**DOI:** 10.1038/s41523-021-00344-3

**Published:** 2021-10-20

**Authors:** Stacey E. P. Joosten, Marius Wellenstein, Rutger Koornstra, Annelot van Rossum, Joyce Sanders, Vincent van der Noort, Maria C. Ferrandez, Rolf Harkes, Ingrid A. M. Mandjes, Hilde Rosing, Alwin Huitema, Jos H. Beijnen, Jelle Wesseling, Paul J. van Diest, Hugo M. Horlings, Sabine C. Linn, Wilbert Zwart

**Affiliations:** 1 Division of Oncogenomics, Oncode Institute, The Netherlands Cancer Institute, Amsterdam, The Netherlands; 2 WSK Medical B.V, Amsterdam, The Netherlands; 3 Department of Internal Medicine and Medical Oncology, Rijnstate hospital, Arnhem, The Netherlands; 4 Division of Molecular Pathology, The Netherlands Cancer Institute, Amsterdam, The Netherlands; 5 Department of Pathology, The Netherlands Cancer Institute, Amsterdam, The Netherlands; 6 Department of Biometrics, The Netherlands Cancer Institute, Amsterdam, The Netherlands; 7 Department of Pharmacy and Pharmacology, Antoni van Leeuwenhoek—The Netherlands Cancer Institute, Amsterdam, The Netherlands; 8 Division of Pharmacology, The Netherlands Cancer Institute, Amsterdam, The Netherlands; 9 Department of Clinical Pharmacy, University Medical Centre Utrecht, Utrecht University, Utrecht, The Netherlands; 10 Division of Pharmacoepidemiology and Clinical Pharmacology, Utrecht Institute for Pharmaceutical Sciences, Utrecht University, Utrecht, The Netherlands; 11 Department of Pathology, Leiden University Medical Center, Leiden, The Netherlands; 12 Department of Pathology, University Medical Centre, Utrecht, The Netherlands; 13 Department of Medical Oncology, The Netherlands Cancer Institute, Amsterdam, The Netherlands; 14 Department of Biomedical Engineering, Eindhoven University of Technology, Eindhoven, The Netherlands

**Keywords:** Predictive markers, Breast cancer, Tumour biomarkers

## Abstract

Window studies are gaining traction to assess (molecular) changes in short timeframes. Decreased tumor cell positivity for the proliferation marker Ki67 is often used as a proxy for treatment response. Immunohistochemistry (IHC)-based Ki67 on tissue from neo-adjuvant trials was previously reported to be predictive for long-term response to endocrine therapy for breast cancer in postmenopausal women, but none of these trials enrolled premenopausal women. Nonetheless, the marker is being used on this subpopulation. We compared pathologist assessed IHC-based Ki67 in samples from pre- and postmenopausal women in a neo-adjuvant, endocrine therapy focused trial (NCT00738777), randomized between tamoxifen, anastrozole, or fulvestrant. These results were compared with (1) IHC-based Ki67 scoring by AI, (2) mitotic figures, (3) mRNA-based Ki67, (4) five independent gene expression signatures capturing proliferation, and (5) blood levels for tamoxifen and its metabolites as well as estradiol. Upon tamoxifen, IHC-based Ki67 levels were decreased in both pre- and postmenopausal breast cancer patients, which was confirmed using mRNA-based cell proliferation markers. The magnitude of decrease of Ki67 IHC was smaller in pre- versus postmenopausal women. We found a direct relationship between post-treatment estradiol levels and the magnitude of the Ki67 decrease in tumors. These data suggest IHC-based Ki67 may be an appropriate biomarker for tamoxifen response in premenopausal breast cancer patients, but anti-proliferative effect size depends on estradiol levels.

## Introduction

Presurgical window studies are gaining traction to investigate the response to drugs in short time frames. In such a context, traditional clinical trial endpoints are not applicable. In breast cancer, a decrease in the percentage of malignant cells that stain positive for the nuclear proliferation marker Ki67 is frequently used as a proxy for clinical response. Immunohistochemical assessment of Ki67 has been highly debated with concerns regarding inter-institutional variation in staining as well as inter-observer (pathologist) variability^1–5^. Nonetheless, the marker has been and is extensively used in numerous trials. Ki67 was validated as an informative endpoint in several breast cancer trials treating patients with a neoadjuvant aromatase inhibitor (AI) and/or tamoxifen, with predictive value for long-term adjuvant endocrine treatment response^6–10^. Importantly, to our knowledge, these trials focused exclusively on postmenopausal women. Though not previously investigated for validity as an endpoint in premenopausal patients, Ki67 is used in this subpopulation. With a recent recommendation from the FDA to include more premenopausal women in breast cancer trials on hormonal treatment^11^, the use of Ki67 in this subpopulation may increase even further.

We therefore analyzed the performance of Ki67 staining in pre- versus postmenopausal breast cancer patients treated with tamoxifen, enrolled in a neo-adjuvant, endocrine therapy study by comparing the change in IHC-based Ki67 assessed by pathologists and artificial intelligence to gene expression-based Ki67, gene expression signatures capturing proliferation, mitotic figure counts as well as blood levels of tamoxifen or its metabolites and estradiol.

## Results

### IHC-based Ki67 decrease differs between treatment arms

**Fig. 1:**
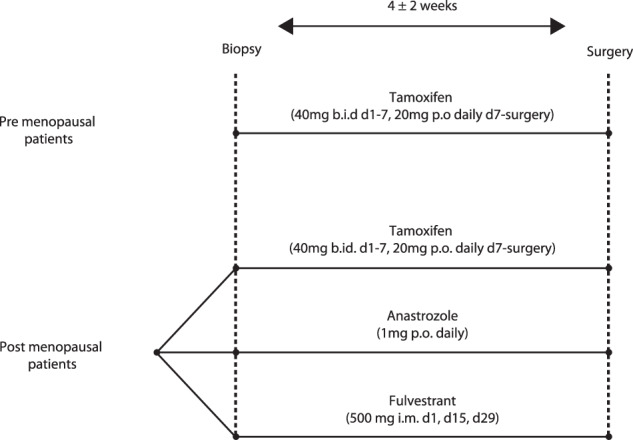
Schematic of trial setup (NCT00738777). Postmenopausal breast cancer patients with ER+ tumors were randomized to several weeks of neo-adjuvant hormonal therapies tamoxifen, anastrozole or fulvestrant. By default, premenopausal patients received tamoxifen. Before the onset of treatment, a biopsy of the tumour was taken, as well as after treatment, during surgery. Time of surgery (thereby treatment duration) was determined by routine clinical planning. b.i.d. = bis in die (twice a day), p.o. = per os (oral), i.m. = intramuscular (injection).

Postmenopausal patients with primary, estrogen receptor-positive (ER+) breast cancer were randomized to several weeks of tamoxifen, anastrozole, or fulvestrant prior to routine surgery, while all premenopausal patients received tamoxifen by default. Tumor material was collected before and after treatment (Fig. 1). The decrease in proliferation in this interval, measured by pathological assessment of IHC-based Ki67, was pre-specified as a primary endpoint. Patient characteristics are described in Table 1 and Supplementary Fig. 1. Treatment duration in premenopausal women was longer at an average of 23.8 days, as opposed to 17.4 days in postmenopausal women receiving tamoxifen (*p* = 0.011), and 19.2 days of treatment among all postmenopausal treatment arms (*p* = NS).

**Table 1:** Baseline characteristics of all patients randomized in the trial and eligible for analysis.

		Premenopausal		Postmenopausal		*P* values	
		tamoxifen (*n* = 35)	tamoxifen (*n* = 18)	anastrozole (*n* = 17)	fulvestrant (*n* = 19)	tamoxifen pre- versus postmenopausal	post-menopausal arms
Age at inclusion		46.7 ± 0.8 (*n* = 35)	61.5 ± 1.8 (*n* = 19)	62.9 ± 2.0 (*n* = 17)	61.8 ± 2.1 (*n* = 18)	*p* < 0.001	*p* = NS
BMI		25.2 ± 0.9 (*n* = 34)	26.7 ± 0.9 (*n* = 19)	26.8 ± 1.4 (*n* = 17)	28.6 ± 1.4 (*n* = 18)	*p* = NS	*p* = NS
Hospital	NKI	32 (91.4%)	16 (84.2%)	13 (76.5%)	15 (83.3%)	*p* = NS	*p* = NS
	Nijmegen	3 (8.6%)	3 (15.8%)	4 (23.5%)	3 (16.7%)		
Treatment duration in days (Start HT – OR)		23.8 ± 1.7 (*n* = 32)	17.4 ± 1.9 (*n* = 18)	19.5 ± 2.3 (*n* = 16)	21.2 ± 3.0 (*n* = 15)	*p* = 0.011	*p* = NS
***Histopathology***							
Laterality	Right	19 (54.3%)	8 (42.1%)	5 (29.4%)	9 (50.0%)	*p* = NS	*p* = NS
	Left	16 (45.7%)	9 (47.4%)	12 (70.6%)	9 (50.0%)		
	Bilateral	0 (0 %)	2 (10.5%)	0 (0%)	0 (0%)		
Histology	Ductal	24 (68.6%)	17 (89.5%)	13 (76.5%)	12 (66.67%)	*p* = NS	*p* = NS
	Lobular	6 (17.1%)	2 (10.5%)	3 (17.6%)	3 (16.7%)		
	Mixed Ductal/Lobular	2 (5.7%)	0 (0.0%)	0 (0.0%)	1 (5.6%)		
	Other (Mucinous/ Tubular/ Apocrine)	2 (5.7%)	0 (0.0%)	1 (5.9%)	2 (11.1%)		
Differentiation grade	Good	11 (31.4%)	5 (26.3%)	3 (17.6%)	3 (16.7%)	*p* = NS	*p* = NS
	Moderate	15 (42.9%)	11 (57.9%)	9 (52.9%)	9 (50%)		
	Poor	4 (11.4%)	1 (5.3%)	2 (11.8%)	2 (11.1%)		
	Not assessed	4 (11.4%)	2 (10.5%)	3 (17.6%)	3 (16.7%)		
Tumor size in mm		15.4 ± 1.6 (*n* = 31)	18.4 ± 3.2 (*n* = 18)	15.9 ± 2.6 (*n* = 13)	17.6 ± 2.0 (*n* = 18)	*p* = NS	*p* = NS
Type of surgery	Mastectomy	14 (40.0%)	7 (36.8%)	3 (17.6%)	6 (33.3%)	*p* = NS	*p* = NS
	Wide Local Excision	17 (48.6%)	11 (57.9%)	10 (58.8%)	12 (66.7%)		
	Biopsy	3 (8.6%)	1 (5.3%)	4 (23.5%)	0 (0.0%)		
	None	1 (2.9%)	0 (0.0%)	0 (0.0%)	0 (0.0%)		
LN involvement	Negative	25 (71.4%)	12 (63.2%)	11 (64.7%)	12 (66.7%)	*p* = NS	*p* = NS
	(sub)micrometastases	7 (20.0%)	2 (10.5%)	1 (5.9%)	2 (11.1%)		
	Positive	0 (0.0%)	3 (15.8%)	2 (11.8%)	3 (16.7%)		
	NA or ND	2 (5.8%)	2 (10.5%)	3 (17.6%)	1 (5.6%)		
***IHC on pre treatment biopsy***							
IHC ER in %		89.4 ± 3.2 (*n* = 33)	98.8 ± 1.2 (*n* = 17)	92.5 ± 3.9 (*n* = 16)	97.5 ± 1.0 (*n* = 16)	*p* = 0.004	*p* = NS
IHC PR in %		58.9 ± 6.2 (*n* = 32)	66.4 ± 8.1 (*n* = 18)	49.06 ± 11.2 (*n* = 16)	63.9 ± 9.1 (*n* = 14)	*p* = NS	*p* = NS
IHC HER2	Negative	33 (94.3%)	18 (94.7%)	14 (82.4%)	15 (83.3%)	NA	*p* = NS
	Positive	0 (0.0%)	0 (0.0%)	2 (11.8%)	1 (5.6%)		
IHC KI67 in %	Assessed by pathologist	15.2 ± 2.7 (*n* = 33)	17.1 ± 4.1 (*n* = 15)	11.0 ± 1.9 (*n* = 16)	9.3 ± 2.4 (*n* = 16)	*p* = NS	*p* = NS

Categorical variables are displayed as frequencies and corresponding percentages within the treatment group and p values resulted from two-sided Fishers exact tests. Continuous variables are displayed as mean value ± SEM (*n*) and *p* values resulted from Mann Whitney U tests when comparing pre-versus postmenopausal patients randomized to tamoxifen or Kruskal–Wallis tests when comparing all postmenopausal arms. Two-sided *t*-test and ANOVA (unadjusted *p* value displayed) were performed on log (Ki67 + 1) values. NS = not significant

Paired pathologist’ assessed IHC-based Ki67 results were available for 29 premenopausal patients and 14 postmenopausal patients who received tamoxifen, for 15 patients who received anastrozole treatment and for 13 patients treated with fulvestrant (Supplementary Fig. 1). A decrease in percentage positive tumor cells for IHC-based Ki67 was observed in the premenopausal arm treated with tamoxifen (*p*-value < 0.005) as well as in post-menopausal patients who received tamoxifen (*p*-value < 0.001). Interestingly, the extent of decrease between pre- and postmenopausal patients receiving tamoxifen differed (*p* = 0.021), with a larger effect size observed in tumors from postmenopausal patients. Yet, we noted ~50% of premenopausal patients to have increased (red) or equal (orange) Ki67 levels upon tamoxifen treatment (Supplementary Fig. 2). While slides were stained and assessed centrally (with the exception of 1 slide), they were not assessed by a single pathologist. Due to concern on inter- and intra -observer variability^1–5^, we set out to re-assess the slides more objectively by an artificial intelligence algorithm.

**Fig. 2:**
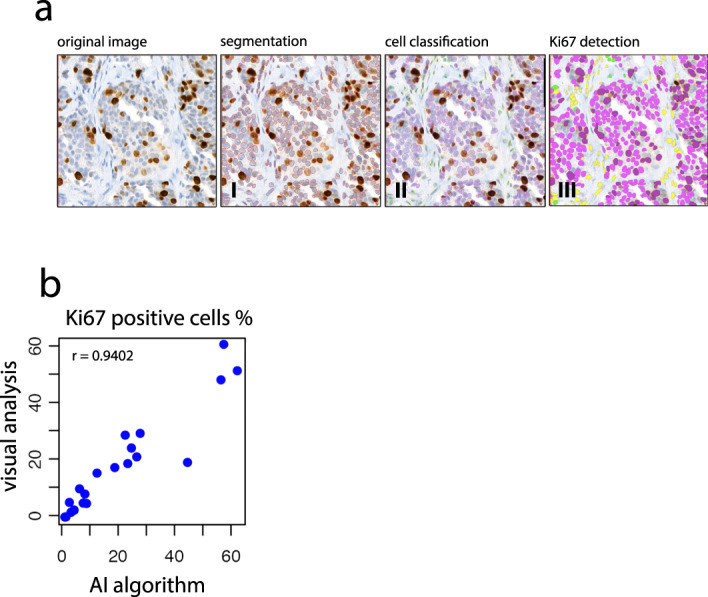
Automated Ki67 scoring by artificial intelligence. **a** Image analysis workflow, including cell segmentation (I), classification of cells (II), labelling as tumor (pink) or stromal (yellow, green), and detection of Ki67 on an individual tumor cell basis (III). **b** Correlation of Ki67% positivity on all individual tumor cells in a 1 mm^2^ hotspot area for each sample (*n* = 20), as assessed by visual inspection versus artificial intelligence algorithm.

A deep learning Ki67 algorithm was developed in collaboration with WSK medical, by means of a convolutional neural network (CNN). The algorithm was trained and validated on a dataset containing whole slide images of KI67 stained tumor tissue of 4599 breast cancer patients treated at the Netherlands Cancer Institute between 2010–2020 (independent from this trial). Whole slide images of breast cancer tissue stained with Ki-67 were retrieved from the Netherlands Cancer Institute Pathology archive. In short, Ki67 staining positivity in tumor cells is determined by the colour and brightness of the staining area within each nucleus contour (Fig. 2a). This resulted in an algorithm with ≥92% accuracy for the detection of positive/negative Ki-67 nuclei (Fig. 2a and b) (detailed description in methods). Before we set out to use this algorithm on all slides from the neo-adjuvant window trial, an expert pathologist marked 1 mm^2^ Ki67-positive tumor hotspots (blinded for treatment arm or timepoint). As a control for the performance of the AI algorithm, hotspot areas for 20 samples from our study were analyzed, in which all individual cells were analyzed on nuclear Ki67 positivity. For this, we used a web-based platform Slide Score (www.slidescore.com) to score and annotate these individual tumor cells, to obtain a percentage of Ki67 positive nuclei of a total number of cells. Each 1mm^2^ hotspot contained more than 1000 tumor cells, which is the recommended minimal amount of tumor cells to assess. Spearman correlation coefficient between the percentage of Ki67 positive tumor cells as assessed by the pathologist versus the AI algorithm was 0.9402 (Fig. 2b). The interclass correlation coefficient between visual analysis and the AI algorithm is 0.942 (ICC) with a 95% confidence interval of 0.863 < ICC < 0.977 (*p*-value; 3.21e^−11^). After establishing the performance of the AI algorithm, we next set out to apply the algorithm on all samples from our study. For 68 out of 71 patients, pre- and post-treatment slides could be retrieved from archives, of which 56 pairs could be assessed by the AI algorithm (Supplementary Fig. 1). On average, 5672 nuclei were counted per pre-treatment slide, versus 5770 per post-treatment slide.

**Fig. 3:**
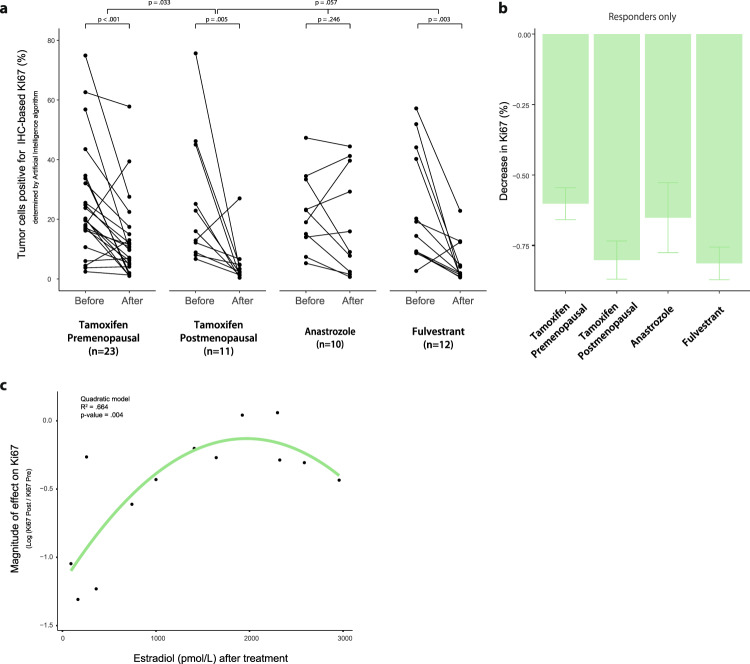
Proliferation in patient tumors assessed by artificial intelligence algorithm. **a** Paired proliferation of breast tumors before and after treatment, assessed by the percentage of tumor cells that stain positive for IHC-based Ki67 determined by an artificial intelligence algorithm. For comparisons between pre- and posttreatment values, per treatment arm, uncorrected 1-sided p values resulting from the Wilcoxon sign ranked test are shown. To evaluate if the treatment effect was larger in premenopausal women who received tamoxifen, as compared to those that were postmenopausal, Mann Whitney U test was performed on the ratios of post- over pretreatment values and the resulting 1-sided *p*-value is displayed. The difference remained significant when only considering patients who decreased in Ki67 levels (e.g. “responders”) with *p* = 0.035. To compare the magnitude of effect among postmenopausal women, the Kruskal-Wallis test was used and the resulting 2-sided *p* value is displayed. **b** Decrease in IHC-based Ki67 levels, as a percentage of pretreatment value, per treatment arm for “responders”. Displayed are mean values per arm, with a standard error of the mean. **c** Relationship between estradiol levels (at time of surgery) for tamoxifen treated women with levels >44 pmol/L and magnitude of effect on Ki67.

The algorithm confirmed that IHC-based Ki67 decreased in both pre- (*p*-value < 0.001) and postmenopausal (*p*-value = 0.005) women who received tamoxifen (Fig. 3a). Increases in Ki67 were notably less frequent as compared to scores from the pathologist reports (Supplementary Fig. 2) and too few per arm for analysis on distinct characteristics. Based on the algorithm results, and in agreement with the pathologist’ observations, the magnitude of Ki67 change still differed between pre- and postmenopausal patients (*p*-value = 0.033) who received tamoxifen. The difference in magnitude remained significant when only comparing patients who decreased in Ki67 levels (i.e. “responders”) (1-sided *p*-value = 0.035). A larger effect size was observed for postmenopausal women with ~63% decrease when considering all patients (or ~80% decrease only considering “responders”) versus ~35% decrease in premenopausal patients (or ~60% decrease when only considering “responders”) (Fig. 3b). In postmenopausal patients, the magnitude of treatment effect on Ki67 did not statistically differ between tamoxifen, anastrozole, and fulvestrant (*p*-value = 0.057), also not when only considering “responders”, which may be impacted by the small sample size in these groups.

To assess whether all tamoxifen-treated patients actually took the drug, serum levels of tamoxifen and metabolites thereof were measured in blood samples taken prior to surgery (Supplementary Fig. 3b). Each patient had detectable tamoxifen values. No differences were found for tamoxifen levels itself between pre- and postmenopausal patients, nor for any of the metabolites we analyzed. None of these variables correlated with the magnitude of the Ki67 change. Duration of treatment, BMI, tumor grade- and histological type did not statistically differ between menopausal status in tamoxifen-treated patients for whom algorithm-assessed Ki67 levels were available (Supplementary Table 1). Treatment duration also did not correlate with the magnitude of effect, when stratified for menopausal status. Lymph node involvement did differ, but even among LN negative patients who received tamoxifen, the magnitude of Ki67 change remained significantly different based on menopausal status. Post-treatment IHC-based PR values (as determined by a pathologist) were slightly higher in postmenopausal patients (*p*-value = 0.001) (Supplementary Table 1), but the effect on PR levels due to tamoxifen was of similar magnitude (*p*-value = NS) in pre- and postmenopausal patients.

We hypothesized that competition of tamoxifen with estradiol, the latter being decreased in menopause, might underlie the difference in treatment effect. We therefore measured estradiol levels (E2) in the blood of patients. For most postmenopausal women, pretreatment E2 levels fell below the detection limit of 44 pmol/l. In premenopausal women, we noted both increases as well as decreases in E2 levels when analyzing pre- and post-treatment E2 measurements (Supplementary Fig. 3a). When we examined the gradient of post-treatment E2 measurements in tamoxifen-treated patients with E2 levels above the detection limit, we found a significant inverse association (*p*-value = 0.004) between the effect size in Ki67 and post-treatment E2 levels (Fig. 3c). Thus, high estradiol levels may compete with tamoxifen to dampen the treatment effect on tumor cell proliferation.

### Gene expression-based Ki67 and proliferation signatures illustrate general decrease of cell proliferation signaling for all treatment arms

Based on the pathological assessment, we found 50% of premenopausal patients to not show a decrease in Ki67 upon tamoxifen treatment. However, assessment by an AI algorithm did not support this. Both methods agreed in the observation that the decrease of Ki67 upon tamoxifen treatment was of lower magnitude for pre-menopausal patients. To provide an independent quantifiable readout of cellular proliferation signaling in relation to treatment response, we generated gene expression data for all arms, as described previously^12^.

**Fig. 4:**
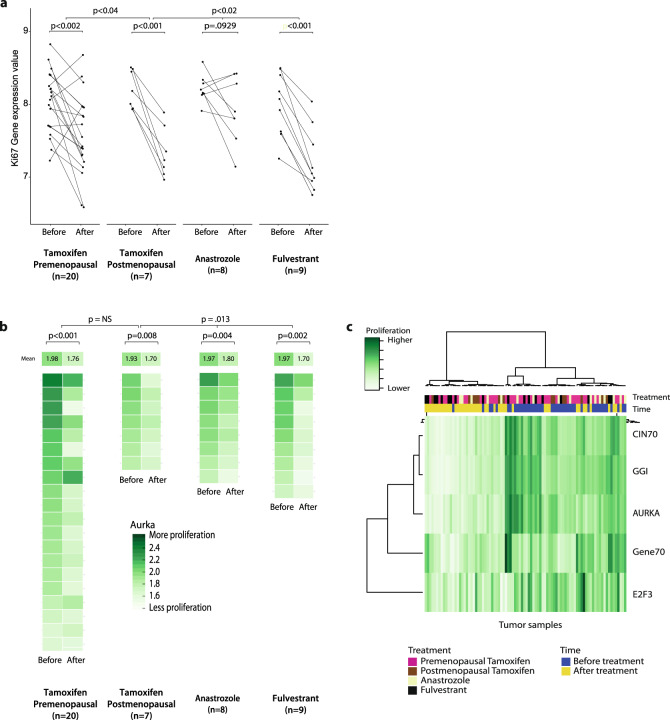
Proliferation in patient tumors assessed by mRNA of Ki67 and gene expression signatures. **a** Paired gene expression values for mRNA of Ki67 of breast tumors before and after treatment. *P*-values resulting from moderated t-tests are displayed for comparisons between before and after treatment, within one arm. To test if the magnitude of effect by tamoxifen was also smaller in premenopausal women on gene expression level, a *t*-test was performed on (post-treatment values - pretreatment values) and the resulting 1-sided *p*-value is displayed. ANOVA was used for comparison among postmenopausal arms. **b** Gene expression signature AURKA, per arm. Each pair of rectangles represent one patient. For statistics, Wilcoxon signed ranked tests were performed and resulting 1-sided *p* values are displayed. To compare the magnitude of effect, Mann Whitney U test was performed for Tamoxifen pre- versus postmenopausal, while Kruskal Wallis was used comparing postmenopausal arms. **c** Heatmap of four additional gene expression-based proliferation signatures.

In almost all cases, mRNA for Ki67 decreased upon treatment (Fig. 4a). When we analyzed the Ki67 mRNA data for a potentially larger anti-proliferative effect in postmenopausal women who received tamoxifen, versus premenopausal patients, we confirmed a statistically significant difference (*p*-value<0.04). To confirm treatment effect on cell proliferation by yet other means, we calculated five independent, previously reported gene expression-based proliferation signatures^13–17^ (Fig. 4b and c, Supplementary Fig. 4, Supplementary Table 2). AURKA signature indicated proliferation decreased statistically significantly in all treatment groups, as did CIN70 and GGI. However, GENE70 scores before and after treatment did not change significantly for premenopausal tamoxifen or anastrozole, nor did E2F3 for anastrozole. Generally, concordance between proliferation signatures was high and unsupervised clustering predominantly aligned with the variable Time (“Before” or “After” treatment), not a treatment or menopausal status (Fig. 4c). The effect size was different when comparing postmenopausal arms amongst each other (*p*-value = 0.013), but on the basis of any signature we were unable to confirm a statistical difference between effect size on proliferation between premenopausal and postmenopausal women receiving tamoxifen (Fig. 4b, Supplementary Fig. 4). Of note, in our dataset, we observed occasional discordance between the proliferation signatures classifying patients as “responders” or “non-responders” (Supplementary Table 2).

As a third independent method to assess cell proliferation, mitotic figures were counted on Hematoxylin and Eosin slides of resection material. In agreement with all above-mentioned readouts, no difference in their number was observed in pre-treatment or post-treatment samples, for all treatment groups (Supplementary Fig. 5).

In general, we found that premenopausal patients as well as postmenopausal patients show a decrease in artificial intelligence algorithm assessed- and mRNA-based Ki67 levels upon tamoxifen treatment in the neo-adjuvant setting. Though, on the basis of both these measurements, the decrease in Ki67 levels was slightly less in premenopausal patients. We found a significant relationship between the magnitude of decrease in Ki67 and patients E2 levels after treatment, which would fit with the biology of tamoxifen competing with estradiol over the binding of the estrogen receptor in these ER+ tumors. Other methods to assess proliferation, such as proliferation signatures and mitotic figure counts, imply that premenopausal patients indeed respond to tamoxifen, but to a similar degree as postmenopausal women.

Taken together, these results imply that IHC-based Ki67, when assessed with rigor, maybe an appropriate biomarker for the premenopausal breast cancer patient to assess tumor response to tamoxifen, on a group level.

## Discussion

IHC-based Ki67 was previously studied and validated as a surrogate endpoint for treatment response to endocrine therapy for ER+ breast cancer. However, none of the postmenopausal trials included a 40 mg tamoxifen loading dose schedule, required to reach steady-state levels within a week^7–10,18^. In addition, these studies were limited to tamoxifen and AI treatment in postmenopausal women. Ki67 is increasingly being used as a response marker for premenopausal women as well, despite a lack of supporting evidence. The number of studies doing so may increase, as the FDA recently issued a guidance document, to encourage the inclusion of more premenopausal women in breast cancer trials that investigate the efficacy of hormonal agents^11^.

We performed a neo-adjuvant trial with postmenopausal women receiving either tamoxifen (including loading dose), anastrozole or fulvestrant, and premenopausal women treated with tamoxifen (including loading dose). Though from a modest number of patients, this dataset allowed us to assess the relationship between response to endocrine treatment on the basis of IHC-based Ki67, within a single patient and per menopausal status, to Ki67 mRNA, proliferation signatures, estradiol levels, tamoxifen metabolites, clinicopathological features, and patient outcome. We were able to confirm, by traditional pathology, that IHC-based Ki67 significantly decreases upon anastrozole treatment, as well as upon tamoxifen treatment in pre- and postmenopausal women. However, in our dataset, initially nearly 50% of premenopausal patients displayed increased or unaltered IHC-based Ki67 levels, which would indicate that these patients did not benefit from the treatment. Increased Ki67 IHC levels in a small subset of patients have been reported in previous studies enrolling postmenopausal patients who received standard-dose tamoxifen^8,9^, arguing against the use of a tamoxifen loading dose as a possible driver for this increase. There has been much debate on inter- and intra- observer variability in IHC-based Ki67, which might also underlie this observation. To more objectively assess the endpoint results of this trial, we employed an artificial intelligence algorithm to detect KI67 levels on immunohistochemistry slides from patient breast tumors. Using this algorithm, we still found IHC-based Ki67 levels to decrease in both pre- and postmenopausal women who received tamoxifen. Assessment of proliferation by other methods, including 5 proliferation signatures and mitotic figures, also support the use of Ki67 as a biomarker for premenopausal women. However, when examining the decrease in IHC-based Ki67 levels as determined by artificial intelligence levels more closely, we noted that the decrease in these was less apparent for premenopausal women who received tamoxifen, than post-menopausal women who received tamoxifen. After excluding this was caused by clinicopathological features such as tumor histology, stage or lymph node involvement, we assessed the magnitude of effect on Ki67 with regards to the gradient of estradiol levels found in the blood of premenopausal women. We found a significant relationship between the two variables, which may reflect competition of estradiol and tamoxifen for the estrogen receptor driving these ER+ tumors. The difference in magnitude of effect in pre- and postmenopausal women was additionally supported by mRNA data. Whether Ki67 effect size corresponds with long-term treatment outcome, is yet to be investigated. This study provides evidence that supports the added value of artificial intelligence in pathology, and may prove useful in addressing the known inter-observer variations in Ki67 scoring, resulting in a highly quantitative measure of Ki67 positivity on IHC. Hence, artificial intelligence may be of use for other neoadjuvant studies using Ki67 IHC as a biomarker for treatment response.

On few occasions, we noted that depending on the method to assess proliferation, a single patient may be considered a “responder” to therapy by one, and a “non-responder” by another method. There may be various reasons that could underlie this, for example - but not limited to – precision of a method (e.g. variance). This includes IHC-based Ki67, for which precision has not been investigated. We therefore advise that interpreting response to therapy in an individual patient should therefore be done with caution. We would like to stress that IHC-based Ki67 was never put forward in literature as a patient-level biomarker nor have patient-level interpretations of IHC-based Ki67 been extensively investigated. Still, clinicaltrials.gov currently lists several breast cancer window trials that utilize IHC-based Ki67 as a primary endpoint measure to assess drug response on a patient level, including those that enroll premenopausal women. In- and outside the context of such trials, patient-level IHC-based Ki67 is used as a measure for endocrine therapy sensitivity and thus treatment guidance. Therefore, we encourage further research into the use of IHC-based Ki67 as a patient-level marker.

To our knowledge, we show the first evidence that supports IHC-based Ki67, when assessed rigorously and quantifiably, can be used as a biomarker to assess tamoxifen response in premenopausal breast cancer patients.

## Methods

### Clinical trial

Between 2008 and 2016, 94 patients with primary, operable, estrogen receptor-positive (ER+ ) breast cancer (Supplementary Fig. 1a for detailed criteria) were registered for an open-label, randomized phase-2 trial (NCT00738777) at the Netherlands Cancer Institute and the Radboud Medical Centre. The primary objective of the trial was to prospectively investigate whether short-term endocrine treatment can induce molecular changes predictive of therapy response. The decrease in proliferation in this interval, measured by pathologist’ assessment of IHC-based Ki67, was pre-specified as a primary endpoint. A core needle biopsy of the tumor was taken prior to treatment. Following treatment, a surgical specimen was taken. The date of the surgery was determined by standard clinical guidelines and planning. All premenopausal women received tamoxifen. Postmenopausal women were randomized to either tamoxifen, anastrozole, or fulvestrant treatment. At the initiation of the trial, fulvestrant was not given as a monotherapy, but combined with anastrozole. After the inclusion of six patients in this arm, a protocol was amended to fulvestrant monotherapy. Dosages were based on previously published studies^19,20^. Of note, a tamoxifen loading dose of 40 mg bi-daily was given in the first week to reach steady-state levels within the duration of treatment^18^.

### Study approval

The clinical trial protocol was approved by the local medical ethics committee of the Netherlands Cancer Institute, in accordance with appropriate international ethical guidelines, and written informed consent was obtained from all patients. The research has been approved by the Netherlands Cancer Institute’s institutional review board.

### Immunohistochemistry and pathologist assessment

Immunohistochemistry (IHC) for Ki67 (Ultraview DAB followed by 32 min antibody retrieval and 32 min incubation with MIB M7240, Dako) was performed at a single pathology facility on pre- and posttreatment patient material. Slides were assessed centrally by an experienced breast pathologist by visual inspection of the whole slide. If pathologists indicated a range of percentage positive cells for Ki67, the highest number was used for analyses. Pathologist’ assessed IHC-based Ki67 is lognormally distributed and contained some 0 values. *T*-test and ANOVA were therefore performed on log10 (Ki67 + 1) values. To examine extent of change in pathologist-assessed values of Ki67 across treatment arms, log10 ((posttreatment Ki67 + 1)/(pretreatment Ki67 + 1)) was compared.

### Artificial intelligence algorithm

A convolutional neural network (CNN), was used to develop the Ki-67 algorithm, which is based on millions of patches sourced from Whole Slide Images (WSI), supplied by the NKI. A sliding window of 80 × 80 pixels is first applied to the IHC stained image, with a stride of 10 pixels, to generate image tiles. Then the trained machine learning model outputs a probability of a nucleus in the centre of a tile, i.e. a probability map with a grid size of 10 × 10 pixels. Then a Gaussian filter is applied on the probability map to obtain a nucleus map and thus a set of nucleus contours can be obtained from the nucleus map, resulting in the location of positive and negatively coloured nuclei with also the percentage amount of positive-coloured nuclei within the Region Of Interest. With these patches, we developed a CNN to make patch-level predictions to detect Ki-67 positive and negative stained nuclei. The reliability of the algorithm was tested against the ground truth. The ground truth is based on an annotated dataset^21^ with which we built the initial algorithm. We then expanded the algorithm using data from the NKI Pathology archive, to clarify the nuances in staining intensity (light blue, light gray/blue), and to segment stroma and other “noise” as background. The deep learning algorithm was trained and validated on a dataset containing 4,599 breast cancer tissue WSI supplied from the NKI Pathology archive. Four thousand 80 × 80 pixel patches were then extracted from each WSI, resulting in 18,396,000, 80 × 80 pixel patches. We used 14,716,800 patches for training and 3,679,200 patches for validation.

During model training, the patch-based classification stage takes as input Ki-67 positive WSI containing breast cancer tissue. We randomly extracted millions of small Ki-67 positive and negative patches from the training set. Following the selection of positive and negative training examples, we trained a supervised classification model to discriminate between these two classes of patches.

The staining positivity is determined by the colour and brightness of the area within each nucleus contour. We implemented a sliding window algorithm in order to identify positive and negative nuclei in each region of interest of 1 mm^2^ for each slide. A Gaussian filter is also applied on the probability map to obtain a nucleus map. Thus, a set of nucleus contours can be obtained from the nucleus map. The performance of the model was tested on the validation dataset and the percentage of reliability is based on the results from the training against the validation, resulting in a reliability of 92% or higher. The output of the Ki-67 module reports the total number of detected nuclei (positive & negative), the number of Ki-67 positive nuclei and the Ki-67 proliferation index (percentage of the total number of detected nuclei that was positive for Ki-67) within the selected region of interest (ROI).

### Tamoxifen, -metabolites and estradiol measurements

Tamoxifen and the five active metabolites N-desmethyltamoxifen, 4-hydroxytamoxifen, 4’-hydroxytamoxifen, N-desmethyl-4-hydroxytamoxifen (Z-endoxifen) and N-desmethyl-4’-hydroxytamoxifen were quantified in patient serum with a validated liquid chromatography-tandem mass spectrometry bioanalytical method^22^. Estradiol measurements were performed using a second-generation Cobas Estradiol immunoassay, and run on a Cobas 6000 device from Roche Diagnostics, following the standard manufacturer’s instructions.

### Gene expression

RNA was isolated and hybridized to a custom full genome array by Agendia as described previously^12^. RNA was isolated from FFPE sections from using the Qiagen RNeasy FFPE kit. 50 ng of total RNA was subsequently reversed transcribed, amplified (Rubicon; C-WTA kit C), labeled with Cy3 (Genomic DNA enzymatic Labeling kit; Agilent Technologies), and purified again (Amicon ultra 30 kDa filters). The labeled cDNA was hybridized to a custom full genome array (based on Agilent Catalog #G2514F) at 65 °C for 17 h, then washed, after which the array was scanned with a dual laser scanner (Agilent Technologies). Feature Extraction software v11.5.1.1 was used to quantify fluorescent intensities and those were normalized using DataPrint software v1.15. Missing values were imputed with knn 10, data were batch corrected for date of RNA extraction using ComBat from the R package sva, and the median value was used in case multiple probes mapped to a single gene. Statistical analysis to compare mRNA levels of Ki67 (moderated *t*-test) was performed with Limma v.3.37.3 in R. For gene expression signatures, per signature, gene expression data was subsetted to genes required for the respective signature and the signature score was calculated, per sample, as was previously described^23^.

### Mitotic figure counts

An expert pathologist in mitotic figures (PvD) blindly scored excision specimens from all treatment groups, according to published guidelines^24^. Since the biopsy samples were limited in total surface area and had a poor representation of intra-tumor heterogeneity, only post-treatment resection slides were analyzed. A cellular region of 0.5 cm × 0.5 cm was analysed for each tumor sample, in which extrapolation was needed for a limited number of cases, without a preference for a particular treatment arm or menopausal status.

### Statistics

For each variable, (normal) distribution was assessed using qqplots and histograms with IBM SPSS Statistics 25. In almost all cases, non-parametric tests were applied or data were log-transformed. Prior to any *T*-test, Levene’s test for variance was performed. For detailed explanations, per variable, please see legends.

### Reporting summary

Further information on research design is available in the Nature Research Reporting Summary linked to this article.

## Supplementary information

Reporting Summary

Supplementary Information

## Data Availability

Gene expression data and accompanying clinical parameters are available on the GEO repository (GSE147271). Additional patients parameters can be made available upon request to the corresponding author.

## Code availability

R scripts (run on R version 3.5.1) regarding gene expression analysis are available upon request to a corresponding author. Details regarding these analysis, including used packages, are specified in the Methods section. Code underlying Ki67 artificial intelligence algorithm is proprietary to WSK Medical B.V., Amsterdam, The Netherlands.

## References

[1] Dowsett M (2011). Assessment of Ki67 in breast cancer: recommendations from the International Ki67 in Breast Cancer working group. J. Natl. Cancer Inst..

[2] Sledge GW (2017). Put some PEPI in your step: Ki67s long road to respectability. J. Clin. Oncol..

[3] Polley M-YC (2013). An international Ki67 reproducibility study. JNCI: J. Natl Cancer Inst..

[4] Yerushalmi R, Woods R, Ravdin PM, Hayes MM, Gelmon KA (2010). Ki67 in breast cancer: prognostic and predictive potential. Lancet. Oncol..

[5] Mengel M (2002). Inter-laboratory and inter-observer reproducibility of immunohistochemical assessment of the Ki-67 labelling index in a large multi-centre trial. J. Pathol..

[6] Ellis MJ (2011). Randomized phase II neoadjuvant comparison between letrozole, anastrozole, and exemestane for postmenopausal women with estrogen receptor-rich stage 2 to 3 breast cancer: clinical and biomarker outcomes and predictive value of the baseline PAM50-based intrinsic subtype—ACOSOG Z1031. J. Clin. Oncol..

[7] Ellis MJ (2008). Outcome prediction for estrogen receptor-positive breast cancer based on postneoadjuvant endocrine therapy tumor characteristics. J. Natl Cancer Inst..

[8] Harper-Wynne CL (2002). Comparison of the systemic and intratumoral effects of tamoxifen and the aromatase inhibitor vorozole in postmenopausal patients with primary breast cancer. J. Clin. Oncol..

[9] Dowsett M (2005). Short-term changes in Ki-67 during neoadjuvant treatment of primary breast cancer with anastrozole or tamoxifen alone or combined correlate with recurrence-free survival. Clin. Cancer Res..

[10] Dowsett M (2007). Prognostic value of Ki67 expression after short-term presurgical endocrine therapy for primary breast cancer. JNCI: J. Natl Cancer Inst..

[11] Premenopausal women with breast cancer: developing drugs for treatment (FDA guidance document). https://www.fda.gov/media/142638/download (FDA, June 2021).

[12] Kastrati, I. et al. The NFkappaB pathway promotes tamoxifen tolerance and disease recurrence in estrogen receptor positive breast cancers. *Mol. Cancer Res.* **18**, 1018–1027 (2020). 10.1158/1541-7786.MCR-19-1082 PMC7335344 32245803

[13] Carter SL, Eklund AC, Kohane IS, Harris LN, Szallasi Z (2006). A signature of chromosomal instability inferred from gene expression profiles predicts clinical outcome in multiple human cancers. Nat. Genet..

[14] Sotiriou C (2006). Gene expression profiling in breast cancer: understanding the molecular basis of histologic grade to improve prognosis. J. Natl. Cancer Inst..

[15] Desmedt C (2008). Biological processes associated with breast cancer clinical outcome depend on the molecular subtypes. Clin. Cancer Res..

[16] van ‘t Veer LJ (2002). Gene expression profiling predicts clinical outcome of breast cancer. Nature.

[17] Bild AH (2006). Oncogenic pathway signatures in human cancers as a guide to targeted therapies. Nature.

[18] Fabian C, Sternson L, El-serafi M, Cain L, Hearne E (1981). Clinical pharmacology of tamoxifen in patients with breast cancer: Correlation with clinical data. Cancer.

[19] Knudsen S (2014). Development and validation of a gene expression score that predicts response to fulvestrant in breast cancer patients. PLOS ONE.

[20] Massarweh S (2011). A phase II neoadjuvant trial of anastrozole, fulvestrant, and gefitinib in patients with newly diagnosed estrogen receptor positive breast cancer. Breast Cancer Res. Treat..

[21] Senaras C (2018). Optimized generation of high-resolution phantom images using cGAN: Application to quantification of Ki67 breast cancer images. PLoS One.

[22] Teunissen SF (2011). Development and validation of a quantitative assay for the determination of tamoxifen and its five main phase I metabolites in human serum using liquid chromatography coupled with tandem mass spectrometry. J. Chromatogr. B.

[23] Gao Q (2014). Effect of aromatase inhibition on functional gene modules in estrogen receptor-positive breast cancer and their relationship with antiproliferative response. Clin. Cancer Res..

[24] van Diest PJ (1992). Reproducibility of mitosis counting in 2469 breast cancer specimens: Results from the Multicenter Morphometric Mammary Carcinoma Project. Hum. Pathol..

