# Supplementary material for: IHC-based Ki67 as response biomarker to tamoxifen in breast cancer window trials enrolling premenopausal women
Source: NPJ Breast Cancer. 2021 Oct 20;7:138. doi: 10.1038/s41523-021-00344-3 (PMC8528844; doi:10.1038/s41523-021-00344-3)
Supplement: Supplementary file 1 — Reporting Summary [file 41523_2021_344_MOESM1_ESM.pdf]

nature portfolio

Corresponding author(s): Horlings, Linn, Zwart

Last updated by author(s): 12 july 2021

Reporting Summary

Nature Portfolio wishes to improve the reproducibility of the work that we publish. This form provides structure for consistency and transparency in reporting. For further information on Nature Portfolio policies, see our Editorial Policies and the Editorial Policy Checklist.

Statistics

For all statistical analyses, confirm that the following items are present in the figure legend, table legend, main text, or Methods section.

n/a	Confirmed	
☐	☒	The exact sample size (n) for each experimental group/condition, given as a discrete number and unit of measurement
☐	☒	A statement on whether measurements were taken from distinct samples or whether the same sample was measured repeatedly
☐	☒	The statistical test(s) used AND whether they are one- or two-sided<br>*Only common tests should be described solely by name; describe more complex techniques in the Methods section.*
☐	☒	A description of all covariates tested
☐	☒	A description of any assumptions or corrections, such as tests of normality and adjustment for multiple comparisons
☐	☒	A full description of the statistical parameters including central tendency (e.g. means) or other basic estimates (e.g. regression coefficient) AND variation (e.g. standard deviation) or associated estimates of uncertainty (e.g. confidence intervals)
☐	☒	For null hypothesis testing, the test statistic (e.g. F, t, r) with confidence intervals, effect sizes, degrees of freedom and P value noted<br>*Give P values as exact values whenever suitable.*
☒	☐	For Bayesian analysis, information on the choice of priors and Markov chain Monte Carlo settings
☒	☐	For hierarchical and complex designs, identification of the appropriate level for tests and full reporting of outcomes
☒	☐	Estimates of effect sizes (e.g. Cohen's d, Pearson's r), indicating how they were calculated

Our web collection on statistics for biologists contains articles on many of the points above.

Software and code

Policy information about availability of computer code

Data collection	for pathology imaging data collection, slidescore (www.slidescore.com) was used. RNA was isolated and hybridized to a custom full genome array by Agendia BV.
Data analysis	For expression analyses, Feature Extraction software v11.5.1.1 was used to quantify fluorescent intensities and those were normalized using DataPrint software v1.15. Missing values were imputed with knn 10, data were batch corrected for date of RNA extraction using ComBat from the R package sva, and the median value was used in case multiple probes mapped to a single gene. Statistical analysis to compare mRNA levels of Ki67 (moderated t-test) was performed with Limma v.3.37.3 in R. . In addition, an AI algorithm was used developed by WSK Medical BV.

For manuscripts utilizing custom algorithms or software that are central to the research but not yet described in published literature, software must be made available to editors and reviewers. We strongly encourage code deposition in a community repository (e.g. GitHub). See the Nature Portfolio guidelines for submitting code & software for further information.

Data

Policy information about availability of data

All manuscripts must include a data availability statement. This statement should provide the following information, where applicable:

- Accession codes, unique identifiers, or web links for publicly available datasets
- A description of any restrictions on data availability
- For clinical datasets or third party data, please ensure that the statement adheres to our policy

Gene expression data are available on GEO repository (GSE147271)

nature portfolio

Corresponding author(s): Horlings, Linn, Zwart

Last updated by author(s): 12 july 2021

Reporting Summary

Nature Portfolio wishes to improve the reproducibility of the work that we publish. This form provides structure for consistency and transparency in reporting. For further information on Nature Portfolio policies, see our Editorial Policies and the Editorial Policy Checklist.

Statistics

For all statistical analyses, confirm that the following items are present in the figure legend, table legend, main text, or Methods section.

n/a	Confirmed	
☐	☒	The exact sample size (n) for each experimental group/condition, given as a discrete number and unit of measurement
☐	☒	A statement on whether measurements were taken from distinct samples or whether the same sample was measured repeatedly
☐	☒	The statistical test(s) used AND whether they are one- or two-sided<br>*Only common tests should be described solely by name; describe more complex techniques in the Methods section.*
☐	☒	A description of all covariates tested
☐	☒	A description of any assumptions or corrections, such as tests of normality and adjustment for multiple comparisons
☐	☒	A full description of the statistical parameters including central tendency (e.g. means) or other basic estimates (e.g. regression coefficient) AND variation (e.g. standard deviation) or associated estimates of uncertainty (e.g. confidence intervals)
☐	☒	For null hypothesis testing, the test statistic (e.g. F, t, r) with confidence intervals, effect sizes, degrees of freedom and P value noted<br>*Give P values as exact values whenever suitable.*
☒	☐	For Bayesian analysis, information on the choice of priors and Markov chain Monte Carlo settings
☒	☐	For hierarchical and complex designs, identification of the appropriate level for tests and full reporting of outcomes
☒	☐	Estimates of effect sizes (e.g. Cohen's d, Pearson's r), indicating how they were calculated

Our web collection on statistics for biologists contains articles on many of the points above.

Software and code

Policy information about availability of computer code

Data collection	for pathology imaging data collection, slidescore (www.slidescore.com) was used. RNA was isolated and hybridized to a custom full genome array by Agendia BV.
Data analysis	For expression analyses, Feature Extraction software v11.5.1.1 was used to quantify fluorescent intensities and those were normalized using DataPrint software v1.15. Missing values were imputed with knn 10, data were batch corrected for date of RNA extraction using ComBat from the R package sva, and the median value was used in case multiple probes mapped to a single gene. Statistical analysis to compare mRNA levels of Ki67 (moderated t-test) was performed with Limma v.3.37.3 in R. . In addition, an AI algorithm was used developed by WSK Medical BV.

For manuscripts utilizing custom algorithms or software that are central to the research but not yet described in published literature, software must be made available to editors and reviewers. We strongly encourage code deposition in a community repository (e.g. GitHub). See the Nature Portfolio guidelines for submitting code & software for further information.

Data

Policy information about availability of data

All manuscripts must include a data availability statement. This statement should provide the following information, where applicable:

- Accession codes, unique identifiers, or web links for publicly available datasets
- A description of any restrictions on data availability
- For clinical datasets or third party data, please ensure that the statement adheres to our policy

Gene expression data are available on GEO repository (GSE147271)

Study protocol	not publicly accessible at this point. While the study describes results from a clinical trial, the results as presented here do not describe the primary endpoint of the trial
Data collection	Between 2008 and 2016, 94 patients with primary, operable, estrogen receptor positive (ER+) breast cancer (Supplemental Figure 1A for detailed criteria) were registered for an open label, randomized phase-2 trial (NCT00738777) at the Netherlands Cancer Institute and the Radboud Medical Centre.
Outcomes	response to treatment is assessed on paired pre- and post-treatment specimens, by change in Ki67 IHC levels, Ki67 mRNA, mitotic figures and cell proliferation gene signatures
