# Supplementary material for: IHC-based Ki67 as response biomarker to tamoxifen in breast cancer window trials enrolling premenopausal women
Source: NPJ Breast Cancer. 2021 Oct 20;7:138. doi: 10.1038/s41523-021-00344-3 (PMC8528844; doi:10.1038/s41523-021-00344-3)
Supplement: Supplementary file 2 — Supplementary Information [file 41523_2021_344_MOESM2_ESM.pdf]

Supplementary Figures and Tables

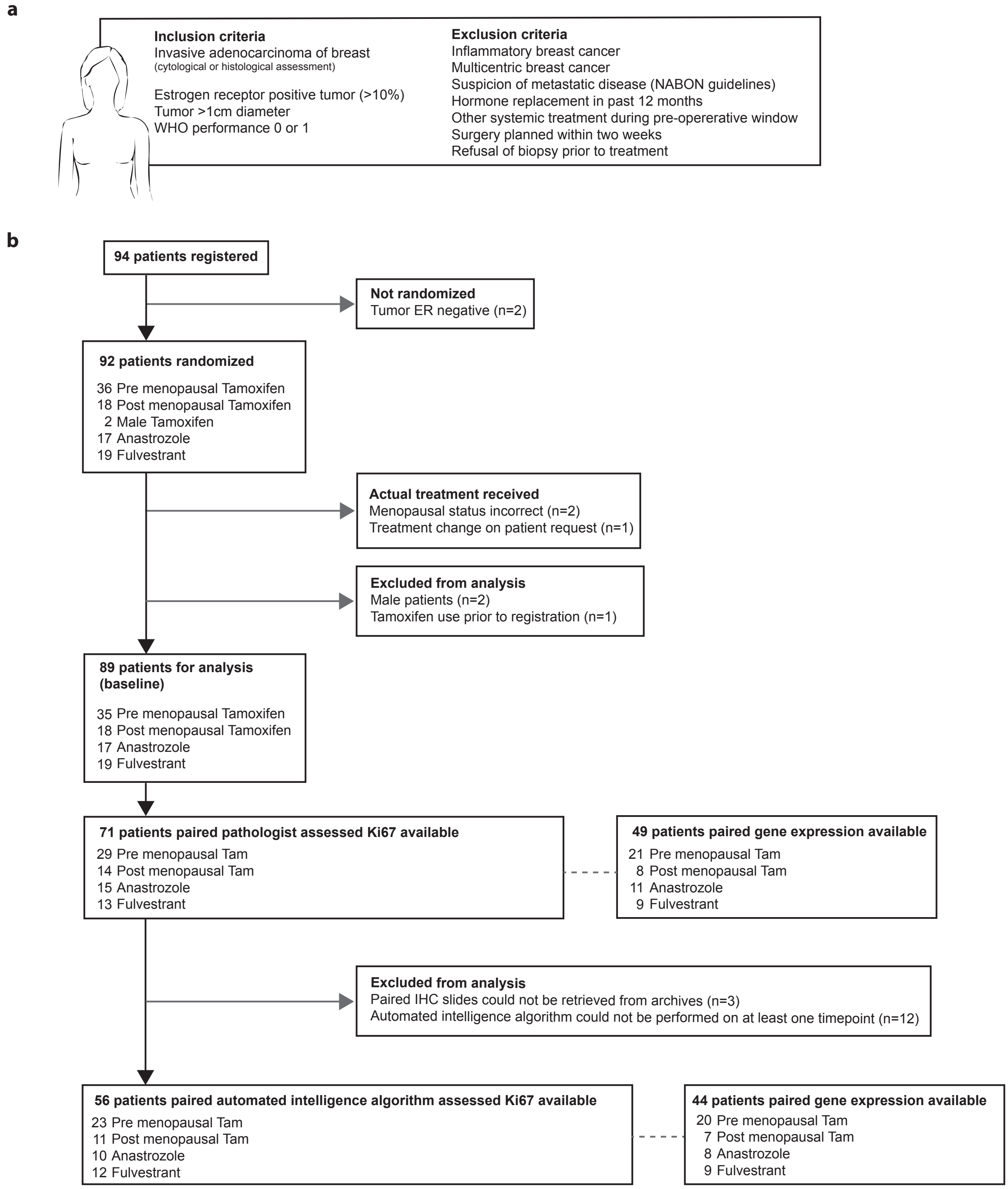

Supplementary Figure 1. Overview of patient inclusion in the trial and those eligible for analyses
a In- and exclusion criteria for NCT00738777.
b Flow diagram of all patients registered and randomized in the trial and those excluded from analyses. Menopausal status of two patients at randomization was incorrect on basis of estradiol blood levels, and one patient refused the treatment she was randomized to. Data from males were excluded from this manuscript due to insufficient numbers. One patient received hormonal therapy prior to start and was therefore also excluded. For 71 out the 89 patients eligible for analysis, paired IHC data on Ki67 was available. For 56 out of those 71 patients, paired automated intelligence algorithm assessed Ki67 values were available from before and after treatment. Out of those, paired gene expression was available for 44 patients.

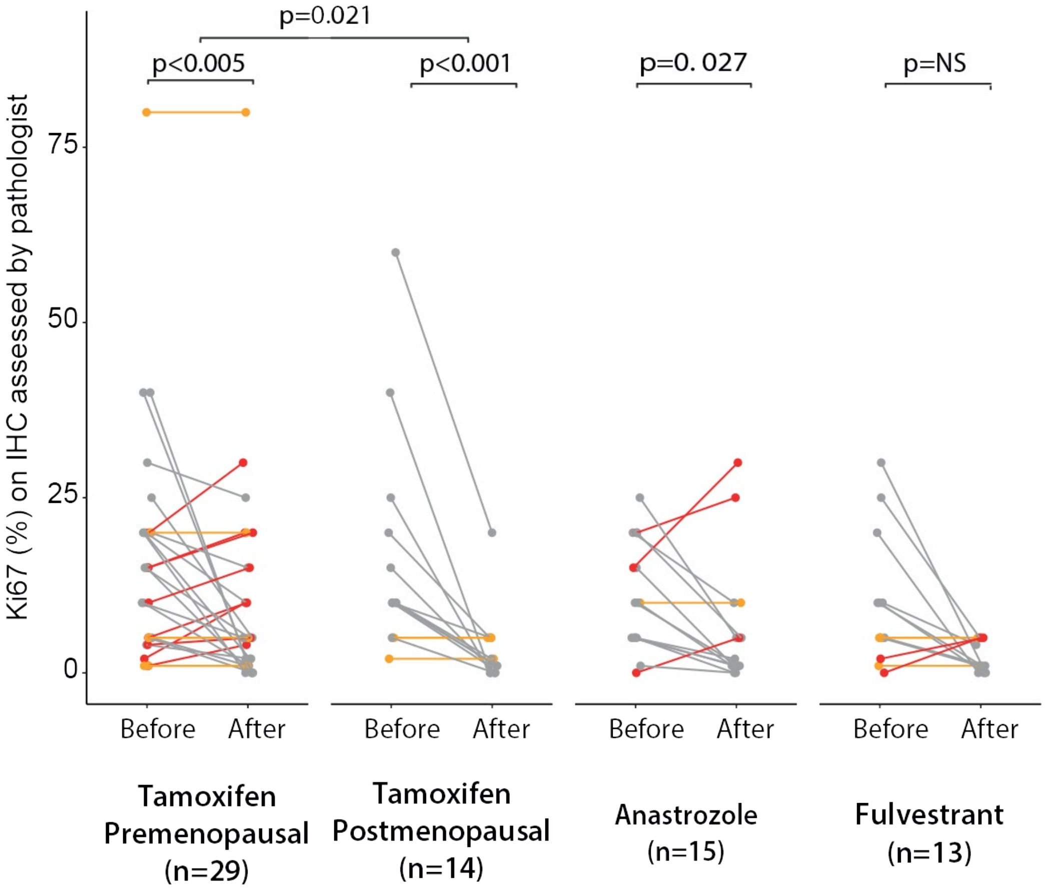

Supplementary Figure 2. Ki67 in patient tumors assessed by pathologist

Paired proliferation of breast tumors before and after treatment, assessed by pathologists for percentage of tumor cells that stain positive for Ki67 on immunohistochemistry. Data from patients with increase in pathologist assessed IHC-based Ki67 are colored in red, those with no change are colored in orange and those with decrease in IHC-based Ki67 are colored in grey. Displayed are uncorrected 1-tailed p values resulting from paired t-tests on log(KI67+1) values or unpaired t-test when comparing between pre- and postmenopausal women who received tamoxifen.

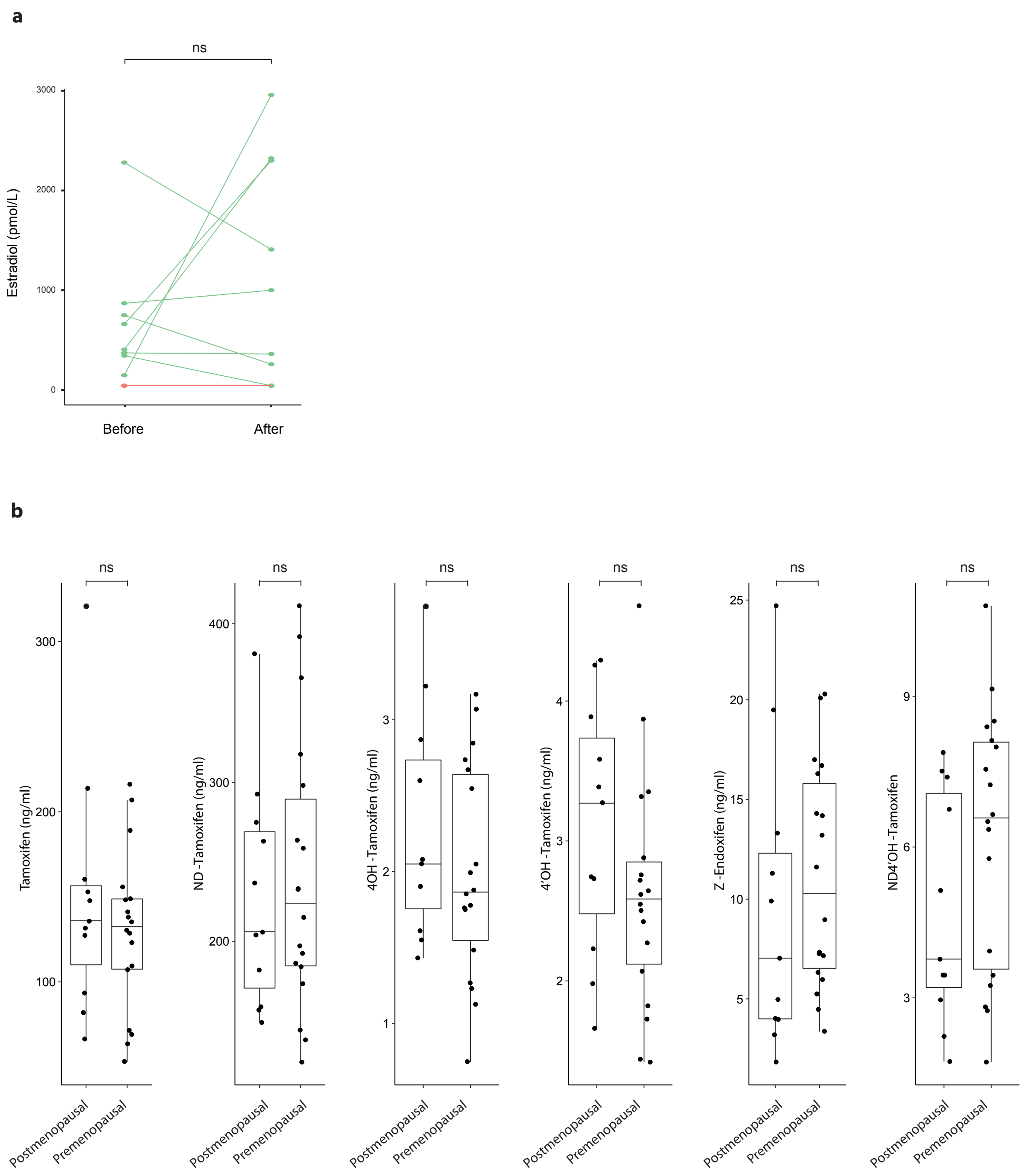

Supplementary Figure 3. Estradiol, tamoxifen and -metabolites blood levels

a Paired estradiol measurements before and after treatment, measured by second generation Cobas Estradiol immunoassay, for premenopausal (green) or postmenopausal (red) Tamoxifen treated patients. E2 levels did not significantly differ between time points, per Wilcoxon paired rank test.

b Measurements of tamoxifen (ng/ml) and a number of its metabolites determined by LC-MS/MS. Two-sided T-tests were performed to compare pre- and postmenopausal levels of 4-OH-TAM, 4’-OH-TAM; Mann Whitney U tests were performed on tamoxifen and ND-4’-OH-tamoxifen; Two-sided T-tests were performed on log transformed values of ND-TAM and Z-endoxifen.

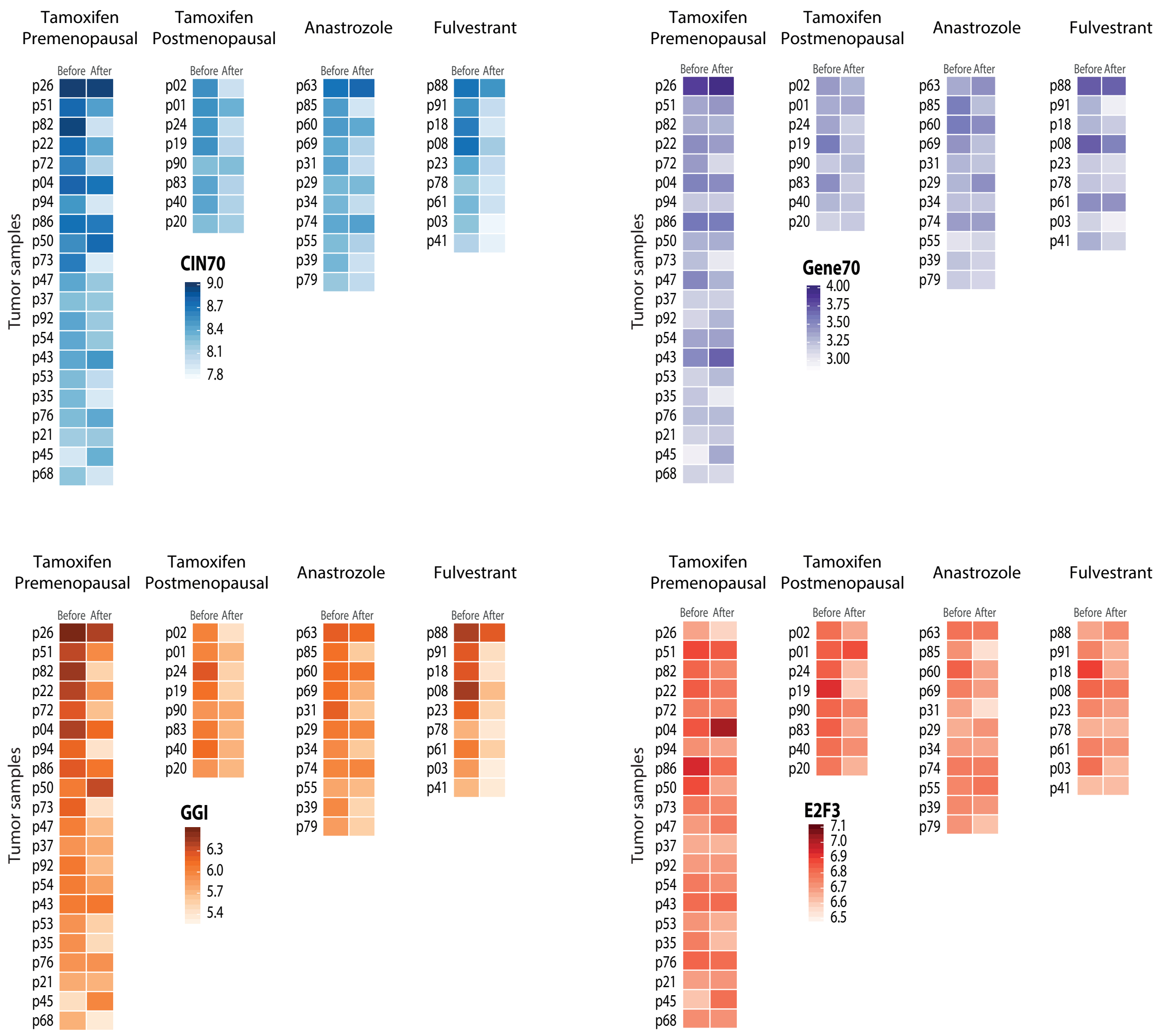

Supplementary Figure 4. Proliferation signatures CIN70, Gene70, GGI and E2F3 on gene expression from patient tumors before and after treatment

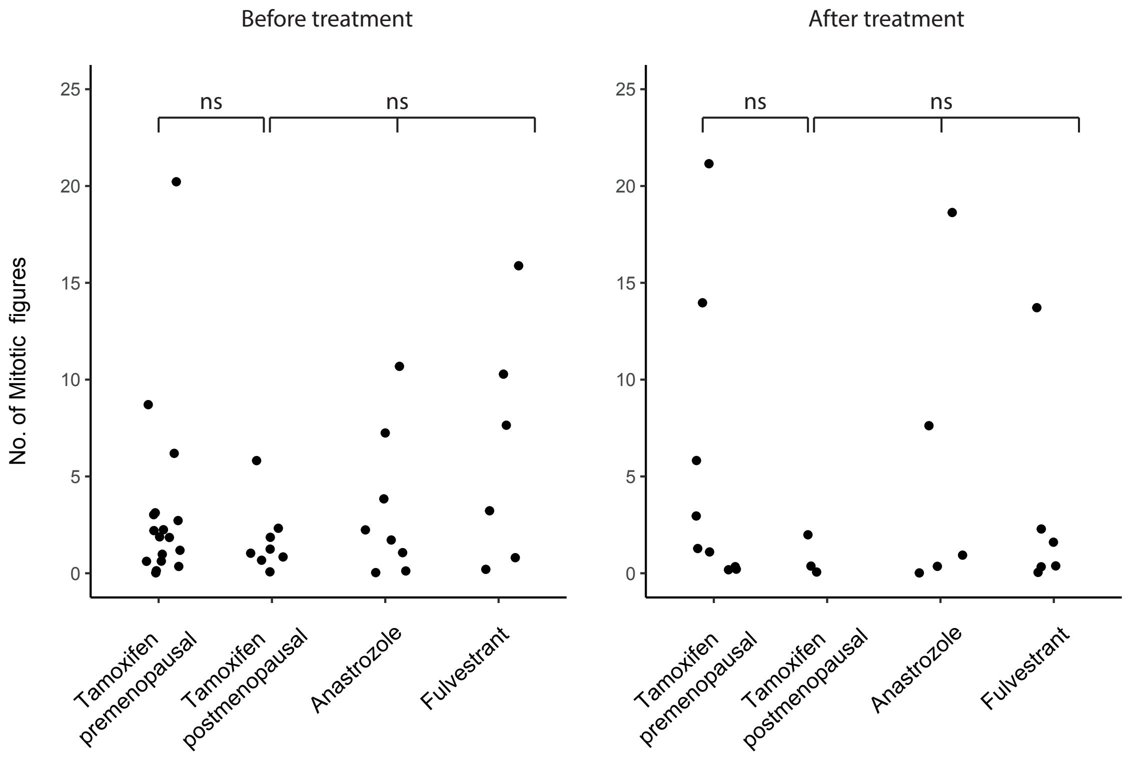

Supplementary Figure 5. Proliferation assessed by mitotic figures Quantification of mitotic figures as assessed by an expert pathologist (PvD). Mann Whitney U tests were performed to compare- pre-versus postmenopausal women who received Tamoxifen, and Kruskal-Wallis tests were performed to compare the postmenopausal patient arm.

		Premenopausal	Postmenopausal			P values	
		tamoxifen (n=23)	tamoxifen (n=11)	anastrozole (n=10)	fulvestrant (n=12)	tamoxifen pre- versus postmenopausal	Post-menopausal arms
Age at inclusion		47.0 ± 1.0 (n=23)	61.1 ± 2.6 (n=11)	64.9 ± 2.0 (n=10)	64.3 ± 2.2 (n=12)	p<.001	p=NS
BMI		26.1 ± 1.2 (n=23)	26.8 ± 1.2 (n=11)	27.1 ± 2.1 (n=10)	28.9 ± 1.3 (n=12)	p=NS	p=NS
Hospital	NKI	20 (87.0%)	8 (72.7%)	7 (70.0%)	10 (83.3%)	p=NS	p=NS
	Nijmegen	3 (13.0%)	3 (27.3%)	3 (30.0%)	2 (16.7%)		
Treatment duration in days (Start HT – OR)		22.6 ± 1.6 (n=23)	17.5 ± 3.0 (n=11)	14.1 ± 2.1 (n=10)	19.9 ± 3.6 (n=12)	p=.081 (NS)	p=NS
Histopathology							
Laterality	Right	11 (47.8%)	3 (27.3%)	2 (20.0%	7 (58.3%)	*p=NS*	*p=NS*
	Left	12 (52.2%)	7 (63.6%)	8 (80.0%)	5 (41.7%)		
	Bilateral	0 (0.0%)	1 (9.1%)	0 (0.0%)	0 (0.0%)		
Histology	Ductal	17 (73.9%)	10 (90.9%)	8 (80.0%)	7 (58.3%)	p=NS	*p=NS*
	Lobular	5 (21.7%)	1 (9.1%)	1 (10.0%)	3 (25.0%)		
	Mixed Ductal/Lobular	1 (4.3%)	0 (0.0%)	0 (0.0%)	1 (8.3%)		
	Other (Mucinous/ Tubular/ Apocrine)	0 (0.0%)	0 (0.0%)	1 (10.0%)	1 (8.3%)		
Differentiation grade	Good	6 (26.1%)	4 (36.4%)	3 (30.0%)	2 (18.2%)	p=NS	*p=NS*
	Moderate	12 (52.2%)	6 (54.5%)	4 (40.0%)	5 (45.5%)		
	Poor	3 (13.0%)	1 (9.1%)	2 (20.0%)	1 (9.1%)		
	Not assessed	2 (8.7%)	0 (0.0%)	1 (10.0%)	3 (27.2%)		
Tumor size in mm		16.7 ± 1.9 (n=22)	20.0 ± 5.1 (n=11)	16.9 ± 2.9 (n=9)	18.8 ± 2.4 (n=12)	p=NS	p=NS
Type of surgery	Mastectomy	8 (34.8%)	5 (45.4%)	2 (20.0%)	2 (16.7%)	p=NS	*p=NS*
	Wide Local Excision	14 (60.9%)	6 (45.5%)	7 (70.0%)	10 (83.3%)		
	Biopsy	1 (4.3%)	0 (0.0%)	1 (10%)	0 (0.0%)		
LN involvement	Negative	17 (73.9%)	5 (45.5%)	7 (70.0%)	8 (66.7%)	p=.023	*p=NS*
	(sub)micrometastases	6 (26.1%)	2 (18.2%)	0 (0.0%)	1 (8.3%)		
	Positive	0 (0.0%)	3 (27.3%)	2 (20.0%)	3 (5.0%)		
	NA or ND	0 (0.0%)	1 (9.1%)	1 (10.0%)	0 (0.0%)		
IHC							
IHC ER in %	Prior to treatment	87.8 ± 4.4 (n=23)	98.2 ± 1.8 (n=11)	91.5 ± 6.1 (n=10)	97.5 ± 1.2 (n=12)	p=NS	p=NS
IHC PR in %	Prior to treatment	55.7 ± 7.2 (n=22)	72.3 ± 10.4 (n=11)	39.5 ± 13.3 (n=10)	68.2 ± 10.9 (n=11)	p=NS	p=NS
IHC PR in %	After treatment	61.7 ± 7.8 (n=23)	95.0 ± 2.4 (n=11)	24.0 ± 7.8 (n=10)	40.5 ± 12.0)	p=.001	p<.001
IHC HER2	Negative	23 (100%)	11 (100%)	8 (80.0%)	11 (91.7%)	p=NS	p=NS
	Positive	0 (0.0%)	0 (0.0%)	2 (20.0%)	0 (0.0%)		
Follow-up							
Recurrence	Nb. of events	4 (17.3%)	3 (27.3%)	0 (0.0%)	2 (16.7%)	p=NS	p=NS

Supplementary Table 1. Characteristics of all patients for whom paired artificial intelligence algorithm scores were obtained.
Categorical variables are displayed as frequencies and corresponding percentages within the treatment group and p values resulted from two-sided Fisher's exact tests. Continuous variables are displayed as mean value ± SEM and p values resulted from Mann Whitney U tests when comparing pre-versus postmenopausal patients randomized to tamoxifen or Kruskal-Wallis tests when comparing postmenopausal arms. Log Rank was used on follow-up data. NS = not significant

Pt_ID	Treatment	KI67_AI	KI67_mRNA	AURKA	CIN70	GGI	Gene70	E2F3
AFT50	Premeno tam	-0.213323307	-0.427981	-0.135042935	-0.199993729	-0.291959212	-0.017878651	0.190732938
AFT60	Anastrozole	-0.206576037	-0.135366	0.09588186	0.075201723	0.056679446	0.10085357	0.154093308
AFT19	Postmeno tam	-0.141054906	1.253235	0.150515502	0.445716555	0.546061049	0.339823709	0.31962402
AFT21	Premeno tam	-0.085677932	0.09596	0.089183935	-0.030119513	0.035339478	-0.050252686	-0.024793795
AFT55	Anastrozole	-0.067380321	-0.15458	0.154262802	0.167690532	0.101221984	-0.083727093	-0.041798209
AFT29	Anastrozole	-0.019261824	0.104596	0.134891704	0.01030479	0.083296462	-0.166985907	-0.055215867
AFT45	Premeno tam	-0.00879916	-0.74788	-0.071167249	-0.434617425	-0.508742533	-0.385922407	-0.186489891
AFT35	Premeno tam	-0.003755668	0.29878	0.24756008	0.387946356	0.429718576	0.200860128	0.135568742
AFT63	Anastrozole	0.028799745	-0.215228	0.234269888	-0.073728137	0.072717049	-0.120118651	0.018501547
AFT24	Postmeno tam	0.031852299	0.475001	0.250886463	0.476033442	0.701563266	0.181599337	0.206505789
AFT39	Anastrozole	0.037085411	1.002791	0.220819965	0.334971618	0.431350826	0.06655786	0.029910908
AFT26	Premeno tam	0.048121022	0.523941	0.23384254	0.017733529	0.191920473	-0.1104885	0.098125245
AFT40	Postmeno tam	0.055203773	0.619279	0.225781356	0.31920251	0.419470957	0.063316558	0.075957981
AFT92	Premeno tam	0.063761022	0.569577	0.276268893	0.231511817	0.381903815	-0.151766814	-0.003019181
AFT20	Postmeno tam	0.064299599	1.041843	0.137642035	0.114688109	0.191938543	-0.047935512	0.133390253
AFT03	Fulvestrant	0.072627878	0.872639	0.310297882	0.429904861	0.542173185	0.18830986	0.162437392
AFT23	Fulvestrant	0.072703305	0.64731	0.335229777	0.370137873	0.664572207	0.097845733	0.055310938
AFT41	Fulvestrant	0.081097368	0.306344	0.166769437	0.256938879	0.330175049	0.160637081	-0.00125995
AFT78	Fulvestrant	0.087374757	0.768574	0.249896947	0.264788659	0.338168734	0.067046919	0.011412071
AFT43	Premeno tam	0.104852663	0.446125	0.147695284	-0.090421844	-0.022583957	-0.197018221	-0.000786589
AFT54	Premeno tam	0.10518238	0.449533	0.216964544	0.18809016	0.212206875	-0.01757564	0.043990114
AFT22	Premeno tam	0.117465772	0.939446	0.248232838	0.32001573	0.45925238	0.073994221	0.06647382
AFT08	Fulvestrant	0.124631659	0.729831	0.443580534	0.557075351	0.748577641	0.167857686	0.041149287
AFT53	Premeno tam	0.124666813	0.957263	0.23169188	0.25173987	0.344482283	-0.108806419	0.054587496
AFT34	Anastrozole	0.127014396	0.629245	0.193542256	0.291211676	0.331699087	0.026429709	0.021134032
AFT86	Premeno tam	0.146258839	-0.319672	0.144674575	0.056549287	0.157453576	0.025887895	0.130695255
AFT90	Postmeno tam	0.1480356	0.592149	0.179788762	-0.001960655	0.106173832	-0.06748307	0.068444964
AFT37	Premeno tam	0.150004531	0.306112	0.14146612	0.055741809	0.126593304	0.005433965	0.017051165
AFT47	Premeno tam	0.150892081	0.919295	0.284031463	0.210901164	0.343604011	0.192834035	-0.070116688
AFT31	Anastrozole	0.152911879	0.436617	0.21643742	0.413328729	0.572043196	0.062784628	0.128926162
AFT61	Fulvestrant	0.15676142	0.9334	0.290147103	0.332241446	0.466681103	0.018415535	0.039385121
AFT94	Premeno tam	0.166427078	1.525922	0.535043293	0.574628375	0.748524516	0.007099151	0.041762086
AFT73	Premeno tam	0.19255997	0.318125	0.301283315	0.76068065	0.74805088	0.228624593	0.036031957
AFT02	Postmeno tam	0.208293685	1.408206	0.418845569	0.567648288	0.560218543	0.106178058	0.131868392
AFT51	Premeno tam	0.210488401	0.446502	0.39949853	0.302099413	0.400176158	-0.068340012	0.035539531
AFT69	Anastrozole	0.243266329	0.77575	0.14607306	0.298834509	0.374784484	0.195896081	0.075072526
AFT01	Postmeno tam	0.247487696	0.795699	0.243203849	0.181374779	0.278382228	-0.007223895	-0.031351122
AFT68	Premeno tam	0.317271465	0.912157	0.03577875	0.293817227	0.367838272	0.039055128	0.008314019
AFT82	Premeno tam	0.334178925	1.305514	0.739817781	0.96641809	0.922531462	0.037466988	0.082642549
AFT88	Fulvestrant	0.343435827	0.44879	0.196872988	0.180344383	0.17016556	0.020567279	-0.055307255
AFT18	Fulvestrant	0.392542753	1.37179	0.52827393	0.720066669	0.824066679	0.09858864	0.226222369
AFT91	Fulvestrant	0.40025116	1.022743	0.285087547	0.511801144	0.778758217	0.325228872	0.100632888
AFT04	Premeno tam	0.469393095	0.379042	0.299438266	0.099548281	0.247781446	0.05465093	-0.165730095
AFT76	Premeno tam	0.473753154	-0.028114	-0.063391343	-0.116128306	-0.01193625	-0.013346791	0.015822299

Supplementary Table 2: (Dis)concordance of proliferation readouts for each patient individually. Values were calculated as pretreatment value minus posttreatment value, thus negative read-outs would indicate increased proliferation after treatment.
